# A Long Way From Home: A Rare Case of Cutaneous Metastasis to the Scalp of Hepatocellular Carcinoma

**DOI:** 10.1155/crdm/9965758

**Published:** 2024-12-20

**Authors:** Evan Eggiman, Paarth Dodia, Jesse Dewey, Melissa Munoz-Bishop, Matthew Overton

**Affiliations:** ^1^Department of Dermatology, Campbell University's School of Medicine, Lillington, North Carolina, USA; ^2^Department of Dermatology, Sampson Regional Medical Center, Clinton, North Carolina, USA; ^3^Department of Pathology, Greensboro Pathology Associates, Greensboro, North Carolina, USA; ^4^Department of Dermatology, Dermatology Group of the Carolinas, Concord, North Carolina, USA

**Keywords:** case report, cutaneous metastasis, hepatocellular carcinoma, immunohistochemistry

## Abstract

**Introduction:** Cutaneous metastases of hepatocellular carcinoma (HCC) are uncommon but important to recognize for timely diagnosis and management.

**Case Presentation(s):** We present a case of a 70-year-old man with a history of HCC who developed a painless nodule on the scalp. Histopathological examination and immunohistochemistry confirmed the nodule as cutaneous metastasis of HCC. The patient had previously undergone transarterial chemoembolization and surgery for HCC, with no evidence of disease for a period before presenting with the cutaneous lesion.

**Conclusion:** Cutaneous metastasis of HCC is rare but signifies advanced disease. This case underscores the importance of considering cutaneous manifestations in patients with a history of HCC and highlights the need for routine follow-up and early intervention to improve patient outcomes.

## 1. Introduction

Cutaneous metastases of hepatocellular carcinoma (HCC) are extremely rare. Identifying these manifestations can be challenging since they typically present as painless, rapidly growing nodules that resemble pyogenic granulomas, subcutaneous abscesses, or hemangiomatous neoplasms [1, 2]. Skin metastasis is estimated to occur in 0.6%–10.4% of all cancer patients, constituting 2% of all skin tumors, with cutaneous HCC being even more rare [3].

Despite the low incidence of cutaneous involvement in HCC, physicians should be aware of such manifestations as they can be indicative of advanced disease or metastasis. In this case, we report a manifestation of cutaneous HCC in a 70-year-old man who had been previously diagnosed and treated for HCC in 2018.

## 2. Case Presentation

**Figure 1:**
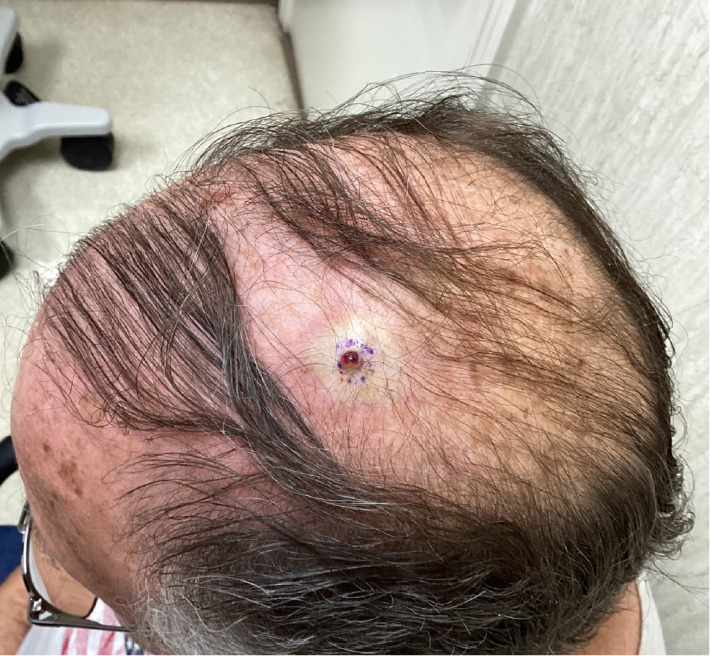
Ulcerative nonpainful nodule on the left lateral scalp.

**Figure 2:**
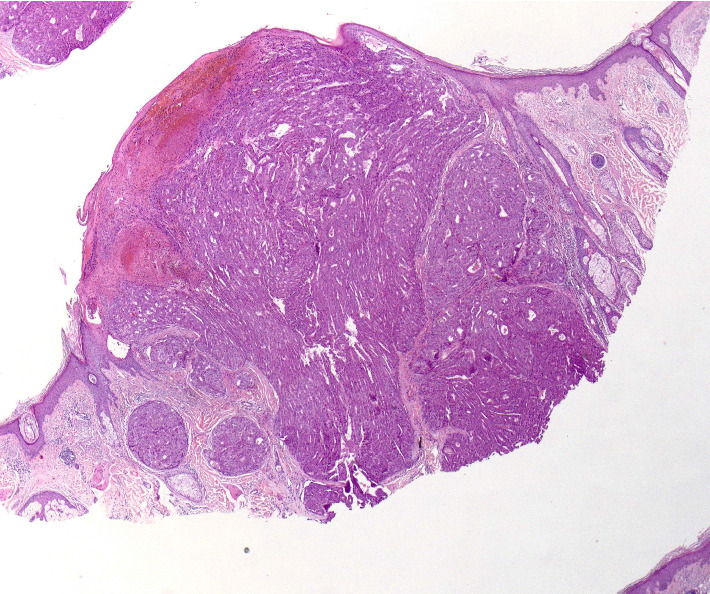
H&E histopathology: low-power view of dermal nodule shows atypical hepatocyte-like cells.

**Figure 3:**
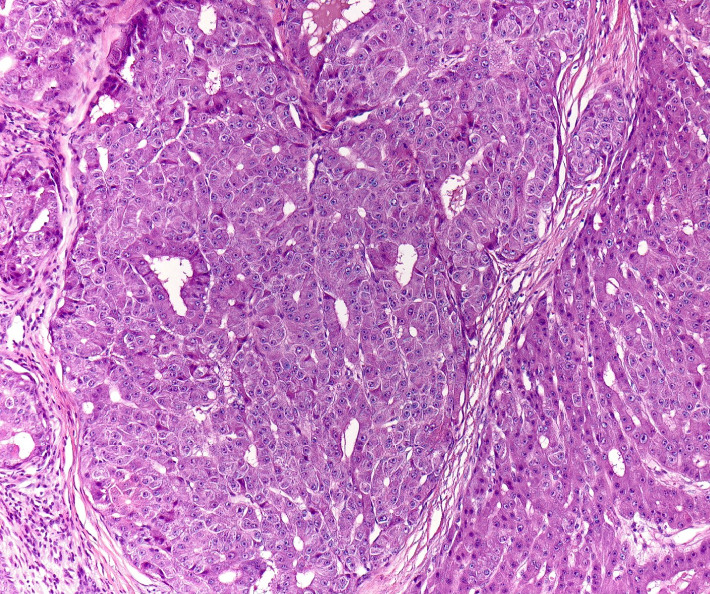
High-power view of dermal nodule shows atypical hepatocyte-like cells resembling densely packed hepatic sinusoids.

**Figure 4:**
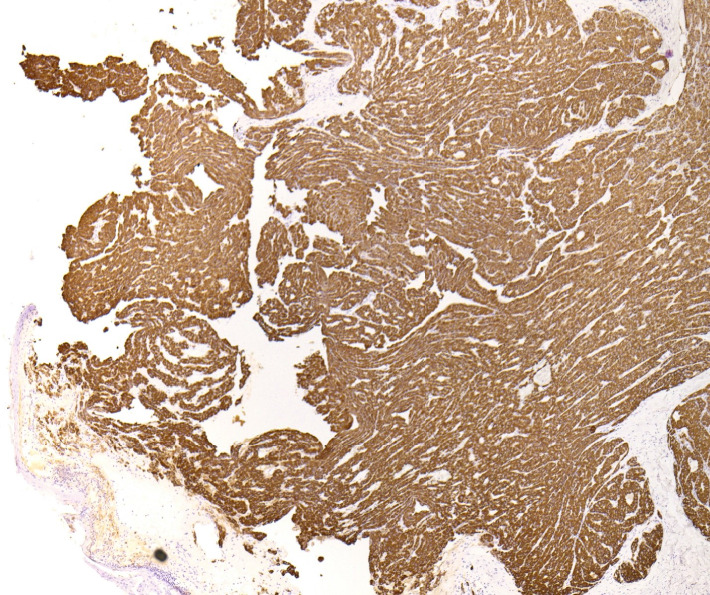
IHC staining shows a uniform presence of Hepar-1.

A 70-year-old man presented to our dermatology clinic with the chief complaint of a nonpainful nodule on the left lateral scalp (shown in Figure 1). A biopsy was performed, revealing a dermal nodule composed of atypical glands with hepatocyte-like cells densely packed in a pattern resembling hepatic sinusoids (shown in Figures 2 and 3). Some of the glandular structures demonstrated bile secretion. Immunohistochemistry was conducted and confirmed the diagnosis of cutaneous HCC as the malignant cells tested positive for CK8/18, hepatocyte paraffin-1 (Hepar-1) (shown in Figure 4), and CD68 showed focal positivity. Notably, the cells were negative for S100, Melan-A, SOX-10, AE1/AE3, CK7, CK20, p63, TTF-1, CD117, EMA, and CDX2. After confirmation of HCC, a treatment plan was made in conjunction with oncology comprising of atezolizumab plus bevacizumab.

The patient had a history of HCC first diagnosed in July 2018. An MRI taken in the same year revealed a 4.8-cm mass in the right hepatic lobe adjacent to the inferior vena cava. One month later, in August 2018, transarterial chemoembolization (TACE) was performed on the HCC to reduce the tumor's size, followed by hepatobiliary surgery in December. The final pathology indicated Grade 2 moderately differentiated tumor confined to the liver, with negative margins and no submitted nodes. The final staging of the tumor was ypT3 ypNx, where the “yp” prefix indicates the staging was performed after neoadjuvant therapy (TACE) and based on the pathologic examination postsurgery. T3 was assigned because there were multiple tumors, with at least one tumor measuring 5 cm across at the time of surgical removal in December. The “Nx” denotes that the regional lymph nodes were not assessed or submitted for pathological evaluation.

In December 2020, the patient underwent an abdominal CT after presenting with a 1.5-cm abdominal subcutaneous nodule. An ultrasound-guided biopsy confirmed HCC, and the lesion was resected in February 2021. The final pathology report confirmed metastatic HCC. The patient was presumed clear of tumor burden after no masses were detected on imaging in February 2023, four months prior to presenting to dermatology with the cutaneous scalp lesion. After the diagnosis of cutaneous HCC metastasis was confirmed, no additional internal evaluations were performed. The oncology team opted to proceed directly with systemic therapy—atezolizumab and bevacizumab—based on the patient's treatment history and the recent imaging results, which had shown no signs of recurrence. The patient did not have any further follow-up with dermatology.

## 3. Discussion

HCC is the most prevalent primary tumor of the liver [4]. Typically, HCC metastasizes to the lungs, bones, and lymph nodes, although instances of metastasis to the skin have been reported [5]. The cutaneous metastasis of HCC may be caused by hematogenous and lymphatic spread or by direct tissue invasion and generally carries a poor prognosis [6]. Because of the nonspecific appearance of the metastatic lesions, immunohistochemistry can be used to confirm the diagnosis with Hepar-1 being the most sensitive and most used [6].

The mechanism of HCC metastasis beyond the liver involves multiple pathways, with some literature discussing iatrogenic causes and seeding. Procedures such as biopsies, percutaneous ethanol injections, radiofrequency ablations, and liver transplantations have been implicated in the seeding of cancer cells, potentially leading to cutaneous metastasis [7]. This seeding phenomenon, characterized by the implantation of malignant cells, is a feared complication in the diagnosis and treatment of HCC, with an incidence rate of up to 2.29% [7]. The patient's history of TACE and subsequent hepatobiliary surgery reflects the possibility of seeding, which may have contributed to the development of his abdominal subcutaneous nodule and metastatic disease.

This case reinforces the need for ongoing and regular follow-up in patients with a history of HCC and highlights the importance of maintaining increased awareness of any new cutaneous lesions in these patients.

## Data Availability

Data sharing is not applicable to this article as no datasets were generated or analyzed during the current study.
